# Network pharmacology-based identification of major component of *Angelica sinensis* and its action mechanism for the treatment of acute myocardial infarction

**DOI:** 10.1042/BSR20180519

**Published:** 2018-11-07

**Authors:** Xiaowei Niu, Jingjing Zhang, Jinrong Ni, Runqing Wang, Weiqiang Zhang, Shaobo Sun, Yu Peng, Ming Bai, Zheng Zhang

**Affiliations:** 1 The First School of Clinical Medicine, Lanzhou University, Lanzhou, Gansu, China; 2 Gansu Key Laboratory of Cardiovascular Disease, The First Hospital of Lanzhou University, Lanzhou, Gansu, China; 3 Department of Internal Medicine, Baiyin Second People’s Hospital, Baiyin, Gansu, China; 4 Department of Cardiovascular Surgery, The First Hospital of Lanzhou University, Lanzhou, Gansu, China; 5 College of Integrated Traditional Chinese and Western Medicine, Gansu University of Chinese Medicine, Lanzhou, Gansu, China; 6 Department of Cardiology, The First Hospital of Lanzhou University, Lanzhou, Gansu, China

**Keywords:** Angelica sinensis, acute myocardial infarction, endoplasmic reticulum stress, network pharmacology

## Abstract

**Background:** To decipher the mechanisms of *Angelica sinensis* for the treatment of acute myocardial infarction (AMI) using network pharmacology analysis. **Methods:** Databases were searched for the information on constituents, targets, and diseases. Cytoscape software was used to construct the constituent–target–disease network and screen the major targets, which were annotated with the DAVID (Database for Annotation, Visualization and Integrated Discovery) tool. The cardioprotective effects of *Angelica sinensis* polysaccharide (ASP), a major component of *A. sinensis*, were validated both in H9c2 cells subjected to simulated ischemia by oxygen and glucose deprivation and in rats with AMI by ligation of the left anterior coronary artery. **Results:** We identified 228 major targets against AMI injury for *A. sinensis*, which regulated multiple pathways and hit multiple targets involved in several biological processes. ASP significantly decreased endoplasmic reticulum (ER) stress-induced cell death both *in vitro* and *in vivo*. In ischemia injury rats, ASP treatment reduced infarct size and preserved heart function. ASP enhanced activating transcription factor 6 (ATF6) activity, which improved ER-protein folding capacity. ASP activated the expression of p-AMP-activated protein kinase (p-AMPK) and peroxisome proliferator-activated receptor γ coactivator 1α (PGC1α). Additionally, ASP attenuated levels of proinflammatory cytokines and maintained a balance in the oxidant/antioxidant levels after AMI. **Conclusion:** *In silico* analysis revealed the associations between *A. sinensis* and AMI through multiple targets and several key signaling pathways. Experimental data indicate that ASP protects the heart against ischemic injury by activating ATF6 to ameliorate the detrimental ER stress. ASP’s effects could be mediated via the activation of AMPK-PGC1α pathway.

## Introduction

Acute myocardial infarction (AMI), a leading cause of morbidity and mortality worldwide, is characterized by cell death resulting from exposure to prolonged ischemia after occlusion of a coronary artery [1]. Timely restoration of myocardial blood flow is a cornerstone of the treatment of AMI. However, reperfusion does not always salvage the ischemic but still viable myocardium, because the time spans for effective reperfusion are short and often the treatment cannot be carried out early enough [1]. Furthermore, reperfusion itself can cause myocardial damage, namely ischemia/reperfusion injury [1]. Therefore, it is clinically important to find an approach to render the heart resistant to ischemia to reduce the loss of cardiomyocytes after AMI.

*Angelica sinensis* (Oliv.) Diels (AS), an ancient Oriental herb, has been used in medicinal prescriptions or nutritional foods in China for thousands of years [2,3]. *A. sinensis* exhibits various pharmacological activities, such as hematopoiesis, antioxidation, anti-inflammation, immunomodulation, anti-apoptosis, and inhibition of platelet aggregation [2,3]. Use of *A. sinensis* alone or in combination with other herbal drugs as traditional Chinese medicine formulae has shown effectiveness in treating anemia, constipation, cardiovascular diseases, hepatic fibrosis, and cancer, while no obvious toxicity was observed at the therapeutic doses [2,3]. Regarding cardioprotective effects, both experimental and clinical studies have highlighted that *A. sinensis* could reduce ischemic injury and improve cardiac function after AMI [2,4,5]. However, due to its extreme complexity both with regard to chemical components and mechanisms of action, it remains poorly understood how *A. sinensis* interacts with the human body. Recently, the emergence of network pharmacology has provided the possibility for a deep understanding of the biological basis for the therapeutic properties of herbal medicines [6]. Network pharmacology is mainly based on large dataset analysis, where the information about herbal active constituents, targets, and diseases is collected to construct constituent–target interactions and target–disease networks [6]. Constituent–target interactions are used to elucidate a molecular mechanism of action of herbs, and target–disease networks are employed to discover the association between diseases and target proteins. Meanwhile, these networks are analyzed using computational methods to identify the major targets of constituents and the related biological processes on a holistic level. A growing body of studies has confirmed the usefulness of the network pharmacology as a tool to unravel the characteristics of multi-constituents, multi-targets, and multi-pathways of herbal drugs [6–8]. Moreover, the predictive results of the network pharmacology could provide clues for future experimental verification [6–8]. Combinations of network pharmacology prediction and experimental verification have become a preferred method to understand the major molecular targets and action mechanisms of herbal drugs [6].

Cardiomyocytes undergo apoptosis by three pathways involving death receptors, mitochondria, and the endoplasmic reticulum (ER) during AMI [9]. ER stress has recently attracted increasing attention because it can be triggered by numerous conditions that cause an imbalance in intracellular homeostasis, such as oxidative stress, hypoxia, and ischemia [10,11]. Initially, ER stress leads to the activation of a series of adaptive processes known as the unfolded protein response (UPR). The UPR induces protective ER-targetted proteins, such as chaperones (e.g. glucose-regulated protein 78 (GRP78), glucose-regulated protein 94 (GRP94), and calreticulin) and protein disulphide isomerases (e.g. protein disulphide isomerase associated 6 (PDIA6)) that restores ER calcium and redox homeostasis and increases ER protein folding capacity [11,12]. However, if these countermeasures are insufficient and severe imbalances persist, ER stress response switches from pro-survival to pro-apoptosis through the activation of C/EBP homologous protein (CHOP) and Caspase-12-dependent pathways [11,12]. The UPR mediates ER stress via three ER-transmembrane sensors: activating transcription factor 6 (ATF6), inositol-requiring enzyme 1α (IRE1α), and pancreatic eIF2-α kinase (PERK). ATF6, a 90-kDa glycoprotein, is maintained in an inactive form (p90-ATF6) in the absence of ER stressors [13]. Upon ER stress, ATF6 is cleaved at the cytoplasmic face of the ER membrane, and the resulting 50 kDa N-terminal fragment of ATF6 (p50-ATF6) translocates to the nucleus, where it activates the expression of ER-targetted genes by binding to the ER stress response elements within their promoter regions [13]. The genes induced by ATF6 are thought to be protective during the acute phase of ER stress, since they encode proteins that are mostly oriented toward re-establishing cellular homeostasis [14]. Ectopically expressed ATF6 has been shown to increase ER-protein folding capacity, and thus aid in the survival of cardiomyocytes during ischemic injury [15–17]. The development of pharmacological strategies that are able to modify dysfunctional UPR to limit severe ER stress may present a promising approach for further treatment of AMI [18,19].

Accordingly, we performed network pharmacology analysis to attain a systematic understanding of the molecular mechanisms of action and the biological activities underlying the therapeutic effects of *A. sinensis* on AMI. Further, *in vitro* and *in vivo* experiments were conducted to confirm the prediction of the *in silico* approach.

## Methods

### Herb constituent collection and target fishing

The herbal constituents and their targets were collected from the TCMSP (Traditional Chinese Medicine Systems Pharmacology) [20], TCM-Mesh (Traditional Chinese Medicine-Mesh) [21], and BATMAN-TCM (Bioinformatics Analysis Tool for Molecular mechANism of Traditional Chinese Medicine) [22] databases. The PolySearch2 [23], an online text-mining system, was also used to obtain information on herb–constituent–target associations. For visualization of the relationship between the constituents and their targets, Cytoscape 3.2.1 software (Cytoscape Consortium, San Diego, CA, U.S.A.) was applied to construct a constituent–target network where constituents and targets were represented with nodes and mapped into interlaced network.

### Candidate AMI-related targets

Candidate AMI targets were obtained from the CTD (Comparative Toxicogenomics Database) [24], TTD (Therapeutic Target Database) [25], GAD (Genetic Association Database) [26], PharmGKB (Pharmacogenomics Knowledgebase) [27], and OMIM (Online Mendelian Inheritance in Man) databases [28].

### Network construction and analysis

Direct and indirect interactions between proteins regulate diverse cellular processes and function in diseases [29]. Therefore, the network including the targets of the collected constituents and second level protein–protein interaction (PPI) data were further constructed using the Bisogenet plugin [30] in the Cytoscape software. The AMI-related target network using the PPI data was also created by the Bisogenet plugin [30]. To unravel the pharmacological mechanisms of *A. sinensis* against AMI, the putative target network and the AMI-related target network were intersected and analyzed using a Cytoscape plugin CytoNCA [31] to calculate topological parameters, including degree centrality (DC), closeness centrality (CC), and betweenness centrality (BC). The nodes with centrality measures that were more than the median centralities of all nodes represent putative major targets of herbs [7,31].

To further clarify the biological roles of the screened major targets, we used DAVID (Database for Annotation, Visualization and Integrated Discovery) [32] to perform the Gene Ontology (GO) biological process enrichment analysis and Kyoto Encyclopedia of Genes and Genomes (KEGG) pathway enrichment analysis. *P*<0.05 was set as the threshold.

### Cell culture and treatment

The rat embryonic ventricular myocardial cell line H9c2 was purchased from the China Center for Type Culture Collection (Beijing, China). Cells were cultured in Dulbecco’s modified Eagle’s medium (DMEM; Gibco, Grand Island, NY, U.S.A.) supplemented with 10% new-born calf serum (Life Technologies, Auckland, New Zealand), 100 U/ml penicillin, and 50 µg/ml streptomycin. Cells were routinely grown in a humidified incubator containing 5% CO_2_ at 37°C to 80% confluence before experiments.

The H9c2 cells were pretreated with 50 µg/ml *A. sinensis* polysaccharide (ASP; Solarbio, Beijing, China) for 4 h and then exposed to oxygen-glucose deprivation (OGD) for 4 h. The interruption of metabolic fuel and oxygen delivery to support cellular metabolism are the cardinal features of ischemia [33]. Cultured cells subjected to hypoxia and fuel deprivation provide an efficient *in vitro* model to examine the cellular mechanisms mediating ischemic injury and cytoprotection [33]. Hypoxic conditions were created in a HeraCell VIOS 160i incubator (Thermo Fisher Scientific, Waltham, MA, U.S.A.) flushed with a gas mixture containing 1% O_2_, 5% CO_2_, and 94% N_2_ at 37°C. During hypoxia, the H9c2 cells were maintained in serum-free and glucose-free DMEM to interrupt supply of the cellular fuel. The corresponding control cells were incubated with the DMEM containing serum and glucose under normoxic conditions for equivalent durations. To further evaluate the effect of AMP-activated protein kinase (AMPK) activation on OGD injury, cells were treated with either specific activator AICAR (500 µM; Selleckchem, Houston, TX, U.S.A.) or inhibitor Compound C (10 µM; Abcam, Cambridge, U.K.) for 4 h before exposure to OGD.

### Flow cytometric analysis

Apoptosis of H9c2 cells was analyzed by flow cytometry using Annexin V-FITC/PI Apoptosis Detection Kit (Multisciences, Hangzhou, Zhejiang, China) according to the manufacturer’s protocol. Briefly, cells were washed twice with cold PBS and resuspended in 100 µl 1× binding buffer. Five microliters of Annexin V-FITC and 10 µl of propidium iodide were labeled with the cells for 5 min in the dark. After incubation, apoptosis was analyzed by flow cytometry (ACEA Biosciences, San Diego, CA, U.S.A.) within 1 h. The percentages of apoptotic cells were calculated as the ratio of the Annexin V-positive cells to the total cells counted. To validate the flow cytometric measurement, H9c2 cells were also pretreated with apoptosis inducers A (Apopisa) and B (Apobid) (Beyotime, Shanghai, China) as positive control.

### RNAi

Specific siRNA for ATF6 and negative control siRNA (siCon) were purchased from GenePharma (Shanghai, China). The sequence of the ATF6 siRNA (siATF6) is sense 5′-GUGUGACUAAACCUGUUCUTT-3′, and antisense 5′-AGACUGAGAACUAGACAACTT-3′. The sequence of the siCon is sense 5′-UUCUCCGAACGUGUCACGUTT-3′ and antisense, 5′-ACGUGACACGUUCGGAGAATT-3′. H9c2 cells were transfected with siRNA using Lipofectamine 2000 (Invitrogen, Carlsbad, CA, U.S.A.) reagent according to the manufacturer’s instructions. To evaluate the specific silencing effect of siATF6, ATF6 expression was detected by immunoblot analysis 72 h post-transfection.

### Animals and treatment

Sprague–Dawley male rats were purchased from the Experimental Animal Center of Lanzhou University (Lanzhou, Gansu, China). The animals were housed under standard laboratory conditions at a room temperature of 22 ± 2°C in a 12-h light/dark cycle with free access to water and food. The study was performed in accordance with the Guide for the Care and Use of Laboratory Animals published by the National Research Council and was approved by the Animal Care Committee of the First Hospital of Lanzhou University.

Rats were anesthetized with chloral hydrate (300 mg/kg) by intraperitoneal injection. Ketoprofen (5 mg/kg) was also administered to minimize post-surgical pain. After tracheal intubation, artificial ventilation was undertaken with a small animal ventilator (Model HX-100E; Chengdu Technology & Market Co., Ltd., Chengdu, Sichuan, China). The chest was opened by a left thoracotomy between the third and fourth ribs. A 6-0 Prolene suture was used to permanently ligate the left anterior descending coronary artery at a point just below the left atrial appendage. The successful induction of AMI was determined by observing a pale ventricle distal to the suture and by ST-segment elevation on the electrocardiogram. Sham rats received the same surgical procedure except for the coronary artery ligation. The animals were randomly assigned to five groups: sham group, AMI group, ASP low-dose group (ASP-L), ASP medium-dose group (ASP-M), and ASP high-dose group (ASP-H). The rats in the three drug treatment groups received ASP through a stomach tube once daily for 4 weeks at a dosage of 100, 200, and 400 mg/kg, respectively. Meanwhile, equal volumes of distilled water were administered intragastrically in the rats in sham and AMI groups.

### Triphenyltetrazolium chloride staining

Myocardial infarct size was assessed by triphenyltetrazolium chloride (TTC) staining. Briefly, the rat hearts were frozen quickly and cut into 2-mm transverse sections. The sections were incubated with 2% TTC in PBS at 37°C for 30 min in the dark and then fixed in 10% paraformaldehyde overnight. The infarcted areas were pale white, whereas the survival myocardium was brick-red. The infarct size was calculated using ImageJ 1.51k software (National Institutes of Health, Bethesda, MA, U.S.A.).

### Echocardiography

Left ventricular (LV) function was evaluated by an echocardiographic system with a MyLabFive ultrasound machine (Esaote, Maastricht, Netherlands) equipped with a scanning transducer (Esaote LA-523) with M-mode recordings. The LV internal dimension at end-diastole (LVIDd) and LV internal dimension at systole (LVIDs) were measured by an operator blinded to the treatment groups. As two indicators of LV function, ejection fraction (EF) was determined automatically by the echocardiography software, and fractional shortening (FS) was calculated as ((LVIDd − LVIDs)/LVIDd) × 100.

### ELISA

ELISA kits (NeoBioscience Technology Co., Ltd., Shenzhen, China) were used to measure the levels of inflammatory cytokines, including interleukin (IL)-1β, IL-6, and tumor necrosis factor (TNF)-α in rat serum and supernatants of H9c2 cells. The ELISA 96-well microtiter plates were analyzed using an infinite M200 PRO microplate reader (Tecan, Männedorf, Switzerland).

### Biochemical assessment

The levels of lipid peroxidation product malondialdehyde (MDA) and the activities of antioxidant marker superoxide dismutase (SOD) in cell lysates and tissue homogenates were determined using respective commercial kits (Jiancheng, Nanjing, Jiangsu, China). All procedures were performed according to the manufacturer’s instructions.

### Western blot

After treatments, proteins were extracted from H9c2 cells or heart tissues lysed in a RIPA buffer containing 1% PMSF and 1% phosphatase inhibitor cocktail (Solarbio). The protein concentration was determined using a BCA protein assay kit (Solarbio). Equal protein extracts were analyzed by Western blotting, with primary antibodies against Bax, GRP78, GRP94, and PDIA6 (Abcam, Cambridge, U.K.); Bcl-2 (R&D Systems, Minneapolis, MN, U.S.A.); Caspase-12, ATF6, PERK, and p-PERK (Proteintech Group, Chicago, IL, U.S.A.); CHOP, cleaved Caspase-3, AMP-activated protein kinase (AMPK), and p-AMPK (Cell Signaling, Danvers, MA, U.S.A.); IRE1α, p-IRE1α, and peroxisome proliferator-activated receptor γ coactivator 1α (PGC1α) (Novus Biologicals, Littleton, CO, U.S.A.); and glyceraldehyde-3-phosphate dehydrogenase (GAPDH; ImmunoWay, Plano, TX, U.S.A.). Immunoblots were detected by ECL (Millipore, Billerica, MA, U.S.A.) with anti-mouse or anti-rabbit IgG coupled to horseradish peroxidase as the secondary antibody (ImmunoWay). Gel images were captured using a Universal Hood II (Bio-Rad, Hercules, CA, U.S.A.) and quantitated with the ImageJ 1.51k software.

### Statistical analysis

All data are represented as the means ± S.D. Differences between mean values were determined by a one-way ANOVA followed by Tukey’s post hoc test using GraphPad Prism 7.00 software (GraphPad Software, San Diego, CA, U.S.A.). A two-sided *P*<0.05 was considered statistically significant.

## Results

### Potential pharmacological mechanisms of *A. sinensis*

#### Screening constituents of *A. sinensis* and their target prediction

**Figure 1:**
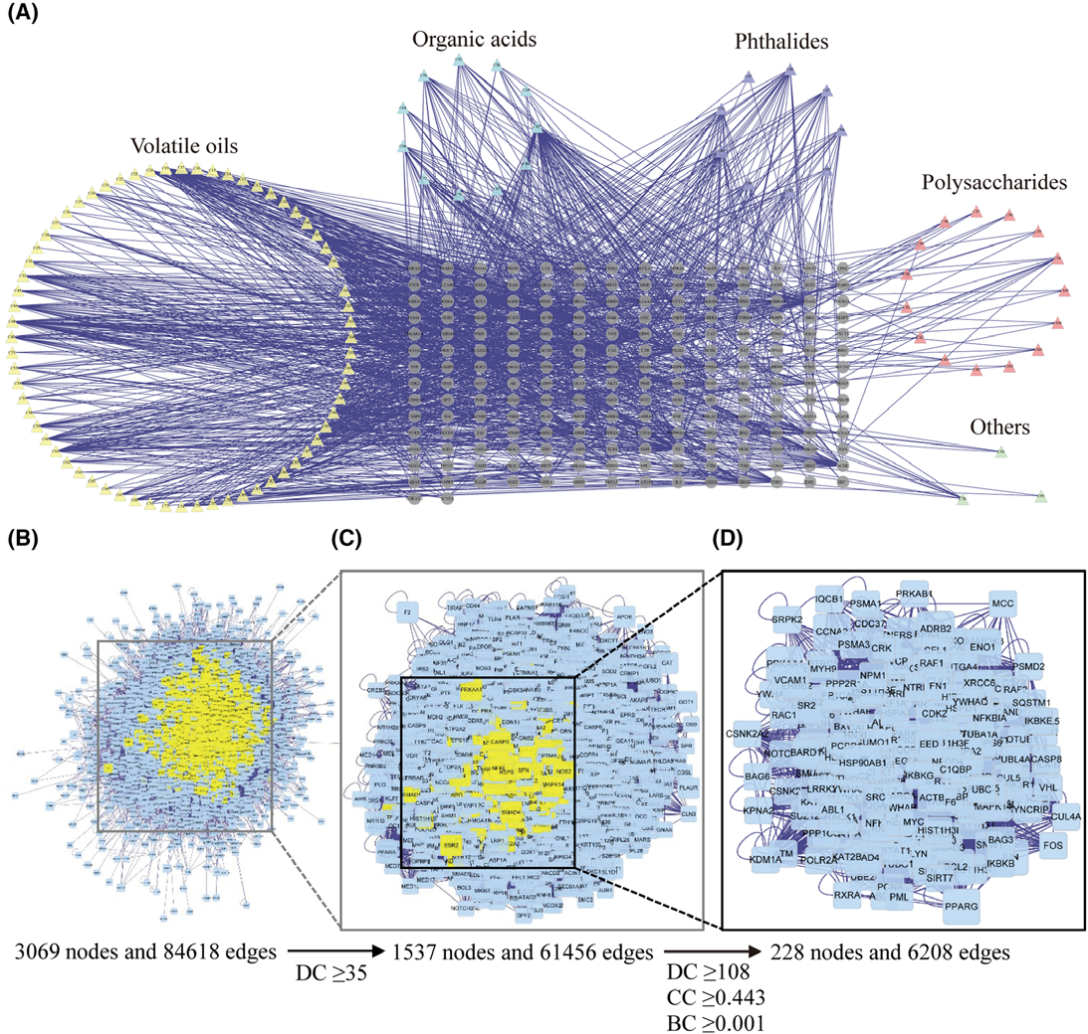
Identification of candidate targets for *A. sinensis* against AMI (**A**) Construction of the constituent–target network of *A. sinensis*. The nodes representing candidate constituents are shown as polychrome triangles and the targets are indicated by gray circle. (**B**) The PPI network including putative targets of *A. sinensis* and known AMI-related targets. (**C**) PPI network of candidate targets extracted from (B). (**D**) PPI network of major targets of *A. sinensis* for AMI treatment extracted from (C).

After the target fishing by the TCMSP, TCM-Mesh, BATMAN-TCM, and PolySearch2 tools, 106 constituents of *A. sinensis* and 1291 targets for these constituents were found. In the constituent–target network (Figure 1A), one constituent may interact with one or more targets and *vice versa* (304 nodes and 1291 edges). These constituents are mainly grouped into four categories: organic acids, volatile oils, phthalides, and polysaccharides. The components that cannot be classified into any of these four classes are assigned into ‘others’.

#### Identification of major targets for *A. sinensis* against AMI

A total of 349 AMI-related targets were collected from five existing resources, namely the CTD, TTD, GAD, PharmGKB, and OMIM. Using the PPI analysis, we constructed a putative target network (3905 nodes and 95987 edges) of *A. sinensis* and an AMI-related target network (6211 nodes and 146902 edges), respectively. Next, the two networks were used to build an intersected network consisting of 3069 nodes and 84618 edges (Figure 1B). Nodes with degrees that were more than the median degree of all nodes were identified as candidate targets. Thus, a network of candidate targets (DC ≥ 35) for *A. sinensis* against AMI was constructed with 1537 nodes and 61456 edges (Figure 1C). Three topological features (DC, CC, and BC) were further selected to identify major targets representing vital roles in AMI treatment. Based on the median values for DC, CC, and BC that were 108, 0.443, and 0.001, respectively, we identified 228 major targets for *A. sinensis* against AMI (Figure 1D).

#### Enrichment analysis of major targets for *A. sinensis* against AMI

Regarding biological function analysis, the significant GO enrichments included negative regulation of apoptotic process, positive regulation of transcription from RNA polymerase II promoter, negative regulation of transcription from RNA polymerase II promoter, regulation of signal transduction by p53 class mediator, Fc-ϵ receptor signaling pathway, stimulatory C-type lectin receptor signaling pathway, positive regulation of transcription, regulation of gene silencing, negative regulation of transcription, G_2_/M transition of mitotic cell cycle, protein stabilization, proteasome-mediated ubiquitin-dependent protein catabolic process, response to drug, cell–cell adhesion, ERBB2 signaling pathway, cellular response to DNA damage stimulus, intrinsic apoptotic signaling pathway in ER stress, cellular response to oxidative stress, T-cell receptor signaling pathway, and DNA damage response.

The KEGG pathway analysis showed that the significant pathways were transcriptional misregulation in cancer, FoxO signaling pathway, estrogen signaling pathway, PI3K-Akt signaling pathway, alcoholism, MAPK signaling pathway, adherens junction, Chagas disease (American trypanosomiasis), proteoglycans in cancer, focal adhesion, ErbB signaling pathway, ubiquitin-mediated proteolysis, B-cell receptor signaling pathway, toxoplasmosis, TNF signaling pathway, apoptosis, endometrial cancer, HIF-1 signaling pathway, Toll-like receptor signaling pathway, and AMPK signaling pathway.

### Experimental validation

On the basis of the above *in silico* analysis, we chose ASP, a major water-soluble component of *A. sinensis*, for further experimental validations because no systematic study has examined the action of ASP against myocardial ischemic injury and the detailed molecular mechanism underlying its cardioprotective role.

### ASP protects H9c2 cells against OGD-induced apoptosis, inflammation, and oxidative stress

**Figure 2:**
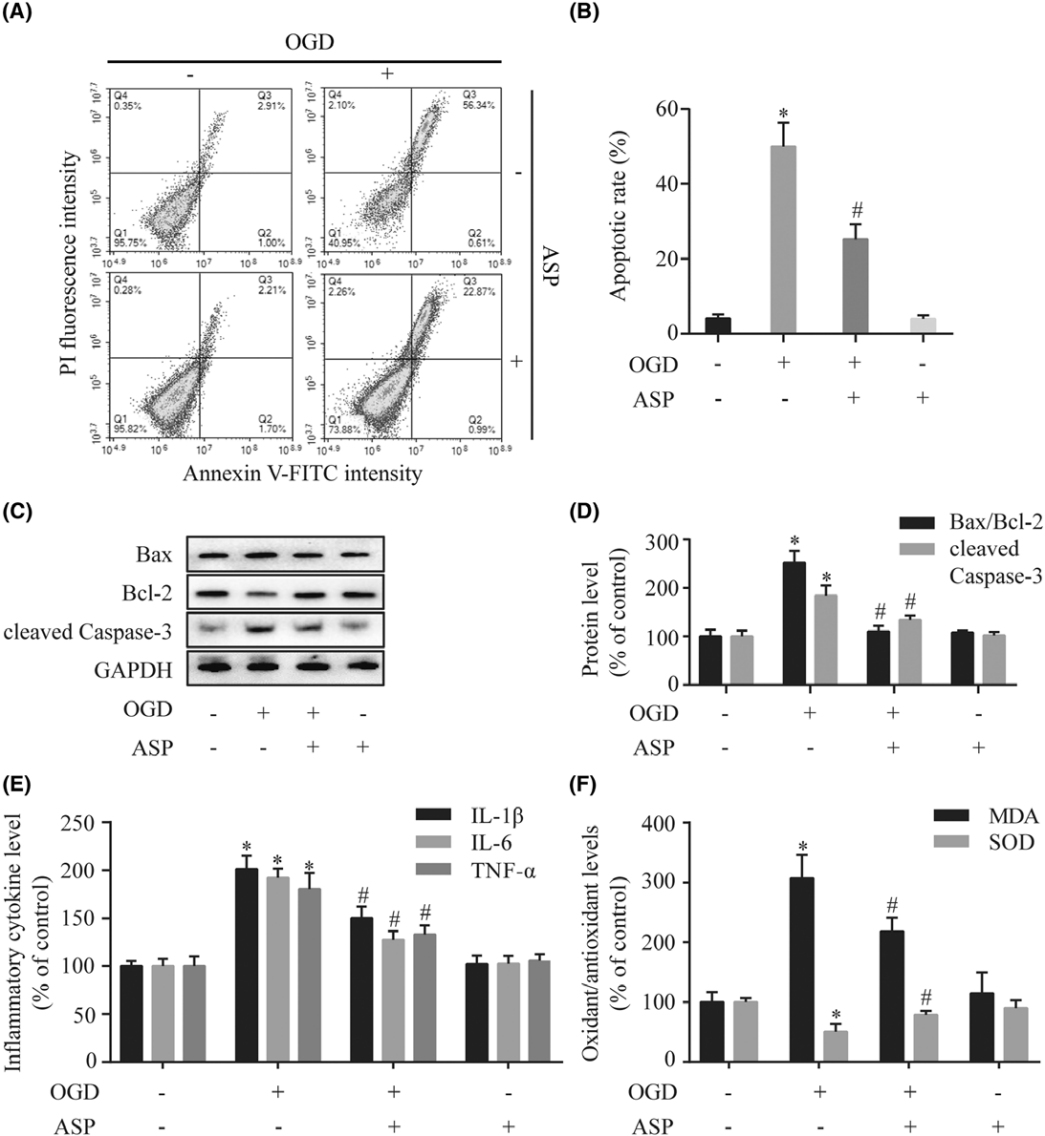
ASP protects H9c2 cells against OGD-induced apoptosis, inflammation, and oxidative stress (**A**) Representative images of flow cytometric analysis by Annexin V-FITC/PI staining. (**B**) Apoptotic cells are represented as the percentage of Annexin V-positive cells. The expression levels of cleaved Caspase-3, Bax, and Bcl-2 were detected by Western blot (**C**), and densitometry (**D**) analyses. (**E**) The levels of proinflammatory cytokines (IL-1β, IL-6, and TNF-α) were analyzed by ELISA. (**F**) The levels of oxidative stress markers (MDA and SOD) were detected by the respective assay kits. *P<0.05 compared with the control group; ^#^*P*<0.05 compared with the OGD-treated group.

To evaluate the effects of ASP on ischemic injury *in vitro*, H9c2 cells were exposed to OGD stress for 4 h in the presence or absence of ASP treatment. Flow cytometric analysis demonstrated that Annexin V-positive cells in ASP pretreatment group were significantly fewer than those in the OGD-treated group (25 ± 4% compared with 50 ± 6%, *P*<0.001) (Figure 2A,B). The results of positive control group demonstrated that our experiments were functioning properly as planned. The regulatory effects of ASP on the expression of apoptosis-associated proteins were examined to confirm the anti-apoptotic effect of ASP on OGD-induced apoptosis. As shown in the Western blot analysis (Figure 2C,D), OGD treatment was associated with a significant increase in the cleaved Caspase-3 level and the Bax/Bcl-2 ratio compared with those in the control group (*P*<0.05). However, pretreatment with ASP significantly decreased the cleaved Caspase-3 expression and Bax/Bcl-2 ratio in OGD-treated H9c2 cells (*P*<0.05 compared with the OGD group). Additionally, we found that ASP treatment alone had no effect on the apoptosis ratio and the expression of the apoptotic regulatory proteins (*P*>0.05 compared with the control group). These results indicated that ASP alleviates apoptosis induced by OGD.

Results of the ELISA showed that OGD treatment significantly increased the levels of IL-1β, IL-6, and TNF-α compared with the control group, while this effect was inhibited by pretreatment with ASP (*P*<0.05 compared with the OGD group) (Figure 2E).

To further evaluate the effect of ASP on OGD-induced oxidative damage, we measured the levels of oxidative stress markers (MDA and SOD). As shown in Figure 2F, OGD injury resulted in an increased production of MDA and decrease in SOD activity. However, pretreatment with ASP significantly attenuated the MDA level and augmented SOD activity in OGD-induced H9c2 cells (*P*<0.05 compared with the OGD group).

### ASP relieves OGD-induced ER stress by activating the ATF6 branch

**Figure 3:**
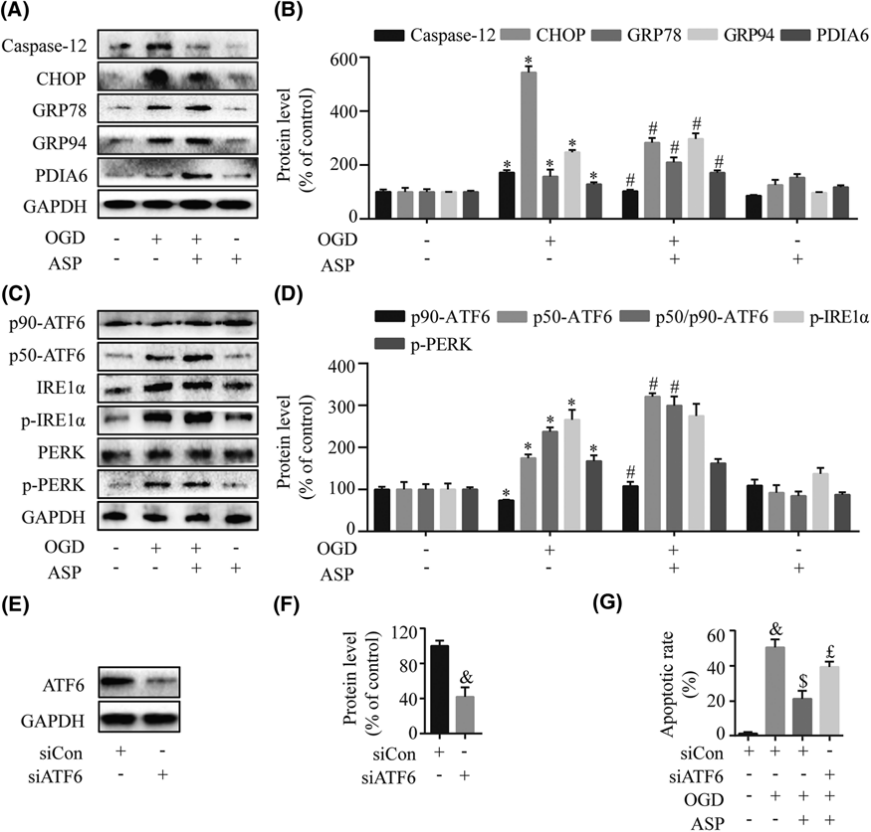
ASP relieves OGD-induced ER stress by activating the ATF6 branch The expression levels of the ER stress-induced pro-apoptosis mediator (CHOP and Caspase-12) and anti-apoptotic molecules (GRP78, GRP94, and PDIA6) were determined by Western blot (**A**) and densitometry (**B**) analyses. The expressions of three transducers in the UPR including ATF6, IRE1α, and PERK were measured by Western blot (**C**) and densitometry (**D**) analyses. (**E**–**G**) H9c2 cells were transfected with siCon and specific siATF6, and then stimulated with OGD for 4 h with or without 50 µg/ml ASP pretreatment. Western blot (E) and densitometry (F) analyses demonstrated a significant knockdown of ATF6 in H9c2 cells after treatment with siATF6. (G) The flow cytometric analysis showed the beneficial effect of ASP was blunted after transfection with siATF6. *P<0.05 compared with the control group; ^#^*P*<0.05 compared with the OGD-treated group; ^&^*P*<0.05 compared with the siCon group; ^$^*P*<0.05 compared with the siCon + OGD group; ^£^*P*<0.05 compared with the siCon + OGD + ASP group.

OGD-induced ER stress was monitored by measuring the expression levels of ER stress-induced pro-apoptosis mediators (CHOP and Caspase-12) and anti-apoptotic molecules (GRP78, GRP94, and PDIA6). Western blot analysis showed that ASP treatment significantly increased the levels of GRP78, GRP94, and PDIA6 while decreasing the expression of CHOP and Caspase-12 compared with the OGD group (*P*<0.05) (Figure 3A,B).

To investigate which UPR signal was involved in the protective effects of ASP against apoptosis induced by the ER stress, we next used Western blot analysis to examine the expression of three transducers in the UPR. The results showed that, in comparison with the OGD group, pretreatment with ASP further enhanced p50-ATF6/p90-ATF6 level (*P*<0.05), while there were no significant effects on phosphorylation of IRE1α and PERK (*P*>0.05) (Figure 3C,D). Taken together, these data suggested that ASP alleviates the destructive ER stress response potentially by selectively activating the ATF6 branch of the UPR.

### ATF6 plays a pivotal role in ASP-mediated cytoprotection

To further determine the role of ATF6 in ASP-mediated protection against OGD injury, we used siRNA to silence the expression of ATF6 in H9c2 cells. Western blot analysis demonstrated a significant knockdown of ATF6 in H9c2 cells (compared with the siCon group, *P*=0.001) (Figure 3E,F). The same results were also found in the flow cytometric analysis (Figure 3G). These observations substantiated the notion that ASP promotes myocardial cell survival through up-regulation of ATF6 expression.

### ASP activates AMPK-PGC1α signaling pathway in H9c2 cells

**Figure 4:**
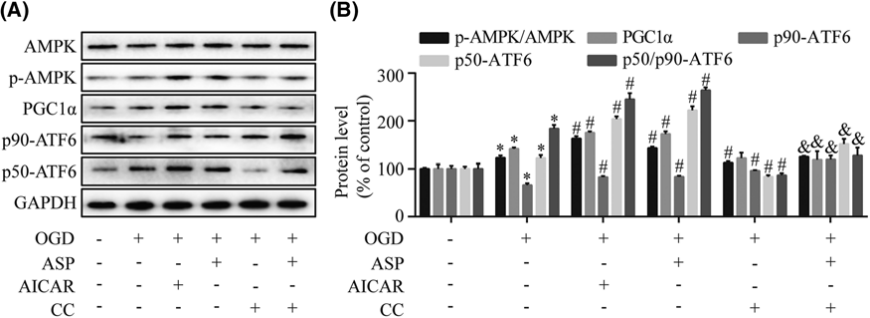
ASP activates AMPK-PGC1α signaling pathway in H9c2 cells H9c2 cells treated with ASP, AICAR (an AMPK activator), or Compound C (CC; an AMPK inhibitor) and then were exposed to OGD for 4 h. The expression levels of total AMPK, p-AMPK, PGC1α, p90-ATF6, and p50-ATF6 were measured by Western blot (**A**) and densitometry (**B**) analyses. *P<0.05 compared with the control group; ^#^*P*<0.05 compared with the OGD-treated group; ^&^*P*<0.05 compared with the OGD + ASP group.

To investigate the mechanism whereby ASP prevents cell death in response to the OGD-induced ER stress, we examined the effects of ASP on the AMPK-PGC1α signaling pathway in stressed H9c2 cells. As shown in Figure 4, ASP pretreatment elevated the levels of p-AMPK, PGC1α, and p50-ATF6/p90-ATF6 in H9c2 cells treated by OGD (*P*<0.05). However, ASP-triggered AMPK activation was suppressed in the presence of Compound C accompanied by reduced levels of PGC1α and p50-ATF6/p90-ATF6. In contrast, we observed that chemical activation of AMPK by AICAR lead to increased expressions of p-AMPK, PGC1α, and p50-ATF6/p90-ATF6. Overall, the AMPK-PGC1α pathway was essential for the effect of ASP on ER stress regulation in H9c2 cells.

### ASP reduces rat myocardial injury after AMI

**Figure 5:**
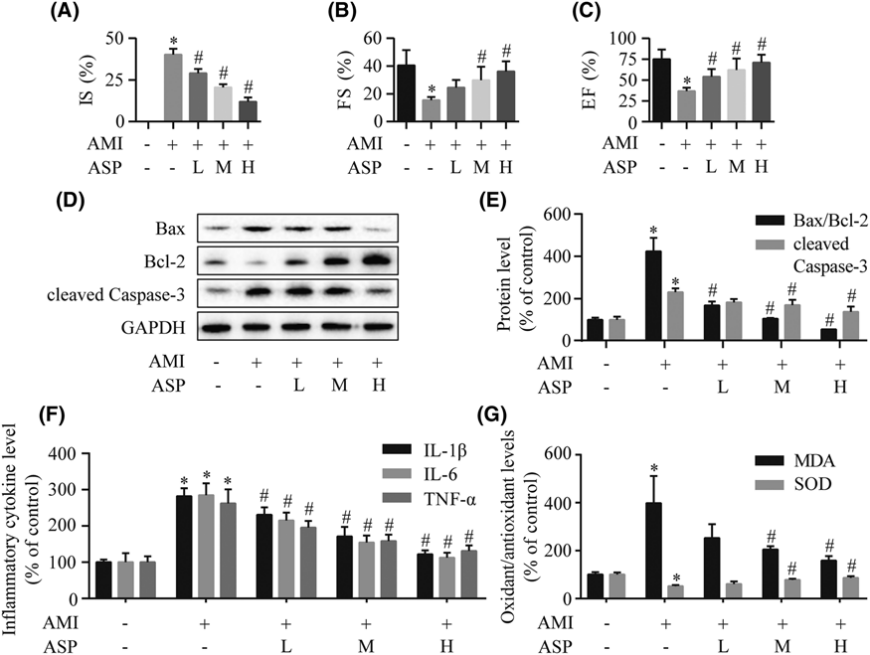
ASP exerts cardioprotection on rats subjected to AMI (**A**) The quantitative analysis of myocardial infarct size (IS) detected by TTC staining. (**B**,**C**) Echocardiographic assessment of cardiac systolic function indicated by LV FS and EF. The expression levels of cleaved Caspase-3, Bax, and Bcl-2 were assessed by Western blot (**D**) and densitometry (**E**) analyses. (**F**) ELISA measurement of IL-1β, IL-6, and TNF-α levels in serum. (**G**) The concentrations of MDA and SOD in myocardial tissue. *P<0.05 compared with the sham group; ^#^*P*<0.05 compared with the AMI group.

To confirm the cardioprotective effects of ASP after AMI, we performed *in vivo* experiments using rats. As shown in Figure 5A, infarct size assessed by TTC staining was smaller in the ASP group than the AMI group. Echocardiographic analysis showed that cardiac function in AMI rats was compromised, as evidenced by significantly reduced EF and FS (Figure 5B,C). However, ASP therapy significantly ameliorated heart dysfunction with increased EF and FS. We further tested the expression levels of the apoptosis-related proteins using Western blot analysis (Figure 5D,E). Cleaved Caspase-3 expression and Bax/Bcl-2 ratio were significantly blocked by ASP treatment in rats. As shown in Figure 5F, we found that pretreatment with ASP significantly decreased the levels of IL-1β, IL-6, and TNF-α compared with the AMI group. Biochemical analyses showed that ASP significantly reversed AMI-induced increase in MDA and decrease in SOD in the heart (Figure 5G). Additionally, the dose-dependent cardioprotective effects could be observed amongst ASP groups. Collectively, our data demonstrated that ASP improves heart function after AMI via the inhibition of apoptosis, inflammation, and oxidative stress.

### ASP attenuates ER dysfunction and activates the ATF6 branch via AMPK-PGC1α pathway in AMI rats

**Figure 6:**
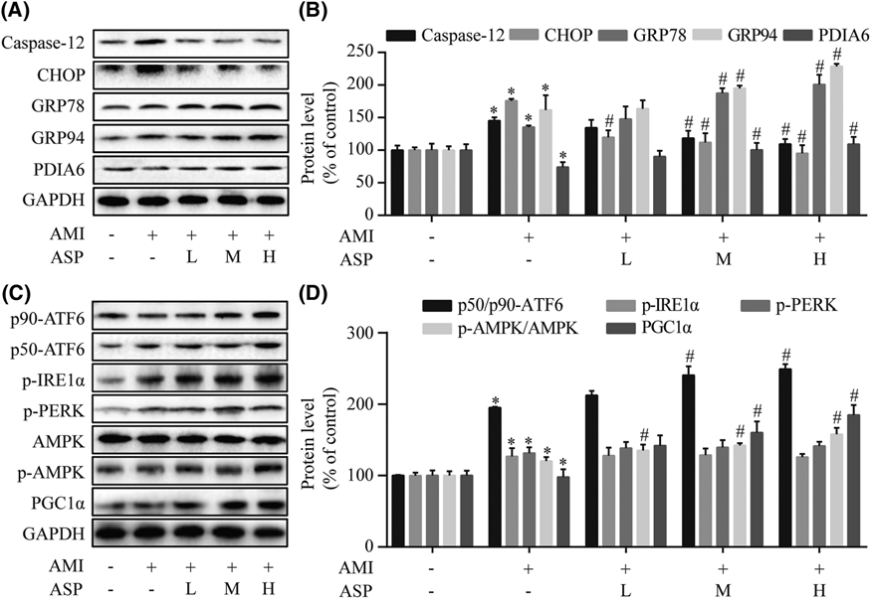
ASP attenuates ER dysfunction and activates the ATF6 branch via AMPK-PGC1α pathway in AMI rats Western blot (**A**) and densitometry (**B**) analyses of marker proteins of the ER stress including CHOP, Caspase-12, GRP78, GRP94, and PDIA6. Western blot (**C**) and densitometry (**D**) analyses of AMPK, PGC1α, and three ER-transmembrane sensors (ATF6, IRE1α, and PERK). *P<0.05 compared with the sham group; ^#^*P*<0.05 compared with the AMI group.

Western blot analysis was used to explore the effects of ASP on ER stress and the underlying mechanism of ASP action in AMI rats. As compared with the AMI group, different doses (100, 200, and 400 mg/kg) of ASP treatment significantly inhibited the expression of CHOP and Caspase-12 but augmented GRP78, GRP94, and PDIA6 levels (*P*<0.05) (Figure 6A,B). We also found that ASP treatment increased AMPK phosphorylation and the levels of PGC1α and p50-ATF6/p90-ATF6 in a concentration-dependent manner (*P*<0.05 compared with the AMI group) (Figure 6C,D). These results indicated that ASP activates the ATF6 branch in ER stressed myocardium of AMI rats via AMPK-PGC1α pathway.

## Discussion

In the present study, network pharmacology analysis was used to explore the major constituents of *A. sinensis* involved in the treatment of AMI. We identified 228 major targets for *A. sinensis* that are central to AMI treatment. Gene enrichment analysis showed that the major targets were involved in several biological processes, such as immune response, cell cycle, apoptosis, protein catabolic process, and oxidative and ER stresses. The most relevant signal transduction pathways that were mapped by the major targets included FoxO, PI3K-Akt, MAPK, ErbB, TNF, HIF-1, and AMPK signaling pathways.

The unknown mechanisms of herbal drugs have become significant obstacles for their further development and internationalization [6]. Network pharmacology is a useful approach to elucidate and predict the inter-relationship between herb interventions and complex diseases [6–8]. Consistent with previous phytochemical studies [2,3], we found that the main chemical components related to the bioactivities and pharmacological properties of *A. sinensis* can be classified as follows: organic acids, volatile oils, phthalides, and polysaccharides. Ferulic acid, the major organic acid present in *A. sinensis*, has been found to exert antioxidant and anti-inflammatory effects against myocardial ischemic injury in rats [34]. Z-Ligustilide and 3-butylidenephthalide, phthalide derivatives from *A. sinensis*, activated antioxidant response element-mediated gene expression and induced anti-inflammatory activities in the heart [35]. The volatile oils of *A. sinensis* possessed anti-inflammatory effects through the inhibition of the secretion of inflammatory mediators [36]. ASP has also been reported to exhibit cardioprotective [37,38] and hepatoprotective [39] activities by ameliorating inflammation, oxidative stress, and apoptosis. These previous studies suggested that multiple constituents of *A. sinensis* may exert a synergistic therapeutic effect against the ischemia-related damages, such as anti-inflammation, antioxidation, and anti-apoptosis. Our current study confirmed and extended these findings using modern computational pharmacological methods, which helped elucidate the underlying pharmacological mechanisms of *A. sinensis* for AMI treatment. Additionally, based on the target prediction analysis, we found that the ER stress may be involved in the protective effects of *A. sinensis* against apoptosis induced by AMI. In the KEGG analysis on the major targets of *A. sinensis*, we found seven signal transduction pathways closely associated with AMI including FoxO, PI3K-Akt, MAPK, ErbB, TNF, HIF-1, and AMPK signaling pathways. A large amount of experimental evidence has accumulated showing that the seven pathways can regulate diverse biological responses in the cardiac context, such as apoptosis, inflammation, oxidative stress resistance, oxygen homeostasis, and energy balance. Our results imply that the constituents of *A. sinensis* may simultaneously target multiple key proteins with functional contributions to the main pathways, which leads the biological system to create a new equilibrium against AMI. AMPK is a crucial serine/threonine kinase that functions as a sensor of cellular energy status in the heart. AMPK activation not only orchestrates a metabolic response to maintain cellular energy status during metabolic stress, but exerts several non-metabolic effects contributing to cell survival under ischemic condition, such as attenuation of ER-associated apoptotic signaling [40,41]. The pharmacological activation of AMPK has been demonstrated to play a key role in reducing myocardial ischemic injury [42]. Given its potent cardioprotection, the AMPK pathway was selected to further validate the efficiency of our computational analyses.

Although many components may contribute to the effects of herbal drugs against different diseases, studying the vital individual constituents is an effective approach to reveal the pharmacological mechanisms of action of herbs [29]. Growing evidence confirms that ER stress is implicated in the regulation of apoptosis under pathogenesis of AMI and is one of contributing factors to ischemic injury [9]. In the present study, we demonstrated that ASP activated the ATF6 branch of UPR and attenuated the detrimental ER stress in OGD-treated H9c2 cells *in vitro* and in ischemic hearts *in vivo*. These activities of ASP were closely associated with the reduced cell apoptosis in response to ischemic injury. The AMPK–PGC1α pathway participated in ASP-mediated protective effects against cardiac injury.

In the setting of AMI, ER stress can be directly initiated by ischemia because protein disulphide bond formation in the ER requires oxygen [10,15]. Moreover, excessive release of free radicals impairs protein folding and post-translational modifications that occur in the ER [43]. ER protein misfolding activates a series of adaptive processes, namely the UPR, to re-establish homeostasis for maintenance of cellular survival. If activation of the pro-survival components of the UPR is insufficient to resolve the stress, persistent ER stress can induce apoptotic or necrotic cell death by pro-apoptotic components, such as CHOP [44] and Caspase-12 [45]. Three ER-transmembrane signaling proteins, IRE1α, PERK, and ATF6, serve as major sensors of the UPR. Activation of the three sensors results in the following responses through their respective target genes: increased expression of ER chaperones and other folding enzymes to enhance the ER protein folding capacity, translational repression of most transcripts to avoid further accumulation of unfolded or misfolded proteins, and degradation of unfolded or misfolded proteins by augmenting the ER-associated protein degradation system. During the processes of UPR, the ATF6 branch is thought to be mainly involved in induction of ER chaperones and enzymes, such as GRP78, GRP94, PDIA6, and other antioxidant proteins, such as catalase [46]. Previous studies have demonstrated that these ER stress-inducible proteins can readjust the ER to cope with stress and exert protective effects in the heart [15,46]. Overexpression of GRP94 protected cardiomyocytes from hypoxia-induced cell death, and preinduction of GRP78 reduced cardiomyocyte necrosis due to oxidative stress [47,48]. Gain- and loss-of-function studies showed that PDIA6 contributed to the protective effects of ATF6 in the ischemic mouse heart by facilitating disulphide bond formation and enhanced ER protein folding [49]. Furthermore, decreased ER-resident protein levels were associated with detrimental accumulation of CHOP and misfolded proteins in the ER, which could result in enhanced cardiomyocyte apoptosis [50]. It has therefore been concluded that the dysfunctional UPR activation after AMI may play an important role in myocardial cell death [11,15]. In the present study, we showed that myocardial ischemia triggered severe ER stress, as evidenced by the increased expression of pro-apoptotic CHOP and Caspase-12 proteins. However, ASP significantly enhanced pro-survival molecules of the ER stress response system, including GRP78, GRP94, and PDIA6 and reduced the pro-apoptotic factors. The results indicated that ASP treatment could recover ER function to limit myocardial ischemic injury.

Several studies have explored the potential of differential regulation of individual UPR branches as strategy for disease treatment. Brief exposure of HeLa cells to a mild temperature (40°C) inducing heat preconditioning activated the PERK branch of UPR and protected cells against apoptosis mediated by hydrogen peroxide [51]. Pharmacological preconditioning with Ginkgolide K preferentially activated the IRE1α branch to limit cardiomyocyte apoptosis induced by tunicamycin, an ER stress inducer [18]. Transgenic mouse models that specifically targetted the ATF6 branch in cardiomyocytes have shown that activation of ATF6 decreased myocardial damage after ischemia and/or reperfusion, while disruption of endogenous ATF6 increased pro-apoptotic signaling and aggravated cardiac function [46,52]. Furthermore, myocardial chronic intermittent hypoxia could induce ATF6 expression to limit infarct size, improve recovery of ventricular systolic function, and protect against ischemia/reperfusion injury, thus increasing cardiac tolerance to acute hypoxic–ischemic injury [53]. These previous findings suggested that ATF6 is required for adaptive ER stress response gene induction and activation of the ATF6 branch of UPR has protective effects during myocardial ischemia [53]. In the present study, we found that ASP acts through activation of the ATF6 branch of UPR to limit ER stress injury both in a cellular model of OGD and in rat models of AMI. Western blot analysis showed that ASP treatment did not considerably affect the phosphorylation of IRE1α and PERK. It might be explained that there were differences in the responses of the three sensors to unfolded proteins and distinct activation kinetics for each sensor [54]. In contrast with the IRE1α and PERK branches, ATF6 activation has been extensively reported to have a cardioprotective role [19]. Our present finding that the beneficial effects of ASP were abolished by siATF6 lends further support to this proposition.

Oxidative stress is a major contributor in myocardial ischemic injury [9,46]. Excessive oxidative stress can cause protein modification, lipid peroxidation, and DNA damage, which is accompanied by activation of an inflammatory response [9]. Treatment with antioxidant agents has demonstrated inhibition of apoptosis and attenuation in myocardial injury [4]. In the present study, we observed that hypoxia/ischemia lead to the imbalance between oxidant and antioxidant levels. Administration of ASP significantly restored SOD activity and reduced the content of MDA. Our results were consistent with previous studies suggesting that the cardioprotective effects of ASP were related to antioxidation activity [37,38].

IL-1β, IL-6, and TNF-α are important proinflammatory cytokines in regulating inflammatory responses [55]. These mediators promote further inflammatory cell adhesion and infiltration into the myocardium, which causes the obstruction of capillary vessels, the production of vasoactive substances, and the release of cytotoxic agents [55]. The disturbed inflammatory stimuli during AMI can aggravate the degree of ischemic injury and delay the recovery of cardiac function [55]. Our results indicated that ASP protected rat from inflammatory insult by occlusion of coronary artery and inhibited OGD-induced inflammatory damage to H9c2 cells. Therefore, suppressing the inflammatory response may be one of the mechanisms by which ASP protects the heart against ischemic injury.

PGC1α plays a central role in the regulation of energy homeostasis, oxidative metabolism, and mitochondrial function in various tissues. Recently, the transcriptional coactivator PGC1α has been shown to concertedly work with ATF6 to regulate different gene expression in response to the ER stress [56,57]. When translocated to the nucleus during the UPR, PGC1α physically interacts with the estrogen-related receptor responsive element in the ATF6 gene promoter, which establishes a positive feed-forward loop inducing ATF6 transcription [56,57]. Pharmaceutical experiments [58,59] have revealed a key role for PGC1α activation in conferring cardioprotective effects. Meanwhile, PGC1α can act as the critical downstream target of AMPK [60]. This is consistent with our observation that inhibition of AMPK significantly down-regulated PGC-1α expression whereas AMPK activation increased PGC1α with the AMPK-specific inhibitor or activator, respectively. We also found that changes in ATF6 expression were consistent with the increase or decrease in AMPK and PGC1α proteins in OGD-treated H9c2 cells. Our *in vivo* data showed that ASP treatment enhanced the expression of AMPK, PGC1α, and ATF6 in a dose-dependent manner. These data suggest that ASP could target the ATF6 arm of the UPR via AMPK-PGC1α signaling, thus ameliorating ischemia-induced injury in the heart.

It should be noted that, although network pharmacology provides useful guidance for identifying potential targets and active constituents of herbal medicines, the target prediction largely depends on the accuracy of prediction tools. Therefore, experimental testing of compound-target binding actions and molecular mechanism of active compounds are required to verify the roles of herbal drugs. The present study has examined only the effects of ASP in the heart. Future studies are needed to test the effects of other constituents of *A. sinensis* on other pathways and their interactions.

## Conclusion

In the present study, we used network pharmacology to study the effective constituents, therapeutic targets, and pharmacological mechanisms of *A. sinensis* for treating AMI. From this network, we inferred the associations between the constituents of *A. sinensis* and AMI through multiple targets and several key signaling pathways. Such *in silico* results were partially confirmed by experimental verification in OGD-treated H9c2 cells *in vitro* and in ischemic hearts *in vivo*. Based on these combined findings, we concluded that ASP selectively activates the ATF6 branch of UPR to ameliorate the detrimental ER stress for treating AMI. The beneficial effects of ASP could be mediated via the activation of AMPK-PGC1α pathway. Additionally, ASP exerts antioxidative and anti-inflammatory effects against myocardial ischemic injury. These findings provide new insight into the cardioprotective effects of ASP and pave the way for further mechanism exploration of other herbal medicines with multiple components.

## Abbreviations

AMI: acute myocardial infarction
ASP: *Angelica sinensis* polysaccharide
ATF6: activating transcription factor 6
Akt: protein kinase B
BATMAN-TCM: Bioinformatics Analysis Tool for Molecular mechANism of Traditional Chinese Medicine
Bax: Bcl2 associated x protein
BC: betweenness centrality
Bcl-2: B-cell CLL/Lymphoma 2
CC: closeness centrality
C/EBP: CCAAT/enhancer-binding protein
CHOP: C/EBP homologous protein
CTD: Comparative Toxicogenomics Database
DC: degree centrality
DMEM: Dulbecco’s modified Eagle’s medium
EF: ejection fraction
eIF2: eukaryotic translation initiation factor 2
ER: endoplasmic reticulum
ERBB2: Erb-B2 receptor tyrosine kinase 2
FS: fractional shortening
FoxO: forkhead box O
GAD: Genetic Association Database
GO: gene ontology
GRP78: glucose-regulated protein 78
GRP94: glucose-regulated protein 94
HIF1: hypoxia inducible factor 1
IL: interleukin
IRE1α: inositol-requiring enzyme 1α
KEGG: Kyoto Encyclopedia of Genes and Genomes
LV: left ventricular
LVIDd: LV internal dimension at end-diastole
LVIDs: LV internal dimension at systole
MAPK: mitogen-activated protein kinase
MDA: malondialdehyde
OGD: oxygen-glucose deprivation
OMIM: Online Mendelian Inheritance in Man
PDIA6: protein disulphide isomerase associated 6
PERK: pancreatic eIF2-α kinase
PGC1α: peroxisome proliferator-activated receptor γ coactivator 1α
PharmGKB: Pharmacogenomics Knowledgebase
PI: propidium iodide
PI3K: phosphatidylinositol 3 kinase
PPI: protein–protein interaction
p-AMPK: p-AMP-activated protein kinase
p50-ATF6: 50 kDa N-terminal fragment of ATF6
RIPA: radioimmunoprecipitation assay
siCon: negative control siRNA
SOD: superoxide dismutase
TCMSP: Traditional Chinese Medicine Systems Pharmacology
TCM-Mesh: Traditional Chinese Medicine-Mesh
TNF: tumor necrosis factor
TTC: triphenyltetrazolium chloride
TTD: Therapeutic Target Database
UPR: unfolded protein response
